# Hybrid Reduced Graphene Oxide with Special Magnetoresistance for Wireless Magnetic Field Sensor

**DOI:** 10.1007/s40820-020-0403-9

**Published:** 2020-03-10

**Authors:** Songlin Yang, Mingyan Tan, Tianqi Yu, Xu Li, Xianbin Wang, Jin Zhang

**Affiliations:** 1 Department of Chemical and Biochemical Engineering, Western University, 1151 Richmond St., London, ON N6A 5B9 Canada; 2 Institute of Materials Research and Engineering, Agency for Science, Technology and Research, Fusionopolis Way, #08-03, Innovis, Singapore, 138634 Singapore; 3 Department of Electrical and Computer Engineering, Western University, 1151 Richmond St., London, ON N6A 5B9 Canada

**Keywords:** Large magnetoresistance, Magnetic nanocrystals, Reduced graphene oxide, Wireless magnetic field sensor

## Abstract

Very few materials show large magnetoresistance (MR) under a low magnetic field at room temperature, which causes the barrier to the development of magnetic field sensors for detecting low-level electromagnetic radiation in real- time. Here, a hybrid reduced graphene oxide (rGO)-based magnetic field sensor is produced by in situ deposition of FeCo nanoparticles (NPs) on reduced graphene oxide (rGO). Special quantum magnetoresistance (MR) of the hybrid rGO is observed, which unveils that Abrikosov’s quantum model for layered materials can occur in hybrid rGO; meanwhile, the MR value can be tunable by adjusting the particle density of FeCo NPs on rGO nanosheets. Very high MR value up to 21.02 ± 5.74% at 10 kOe at room temperature is achieved, and the average increasing rate of resistance per kOe is up to 0.9282 Ω kOe^−1^. In this paper, we demonstrate that the hybrid rGO-based magnetic field sensor can be embedded in a wireless system for real-time detection of low-level electromagnetic radiation caused by a working mobile phone. We believe that the two-dimensional nanomaterials with controllable MR can be integrated with a wireless system for the future connected society.

**Electronic supplementary material:**

The online version of this article (10.1007/s40820-020-0403-9) contains supplementary material, which is available to authorized users.

## Highlights

- Cost-effective and time-efficient process for hybrid reduced graphene oxide (rGO) nanosheets with special magnetoresistance properties was fabricated.
- The prepared hybrid rGO nanosheets have large magnetoresistance at low magnetic field at room temperature and used in wireless magnetic field sensors for quick detection of low electromagnetic radiation.

**Electronic supplementary material:**

The online version of this article (10.1007/s40820-020-0403-9) contains supplementary material, which is available to authorized users.

## Graphical abstract

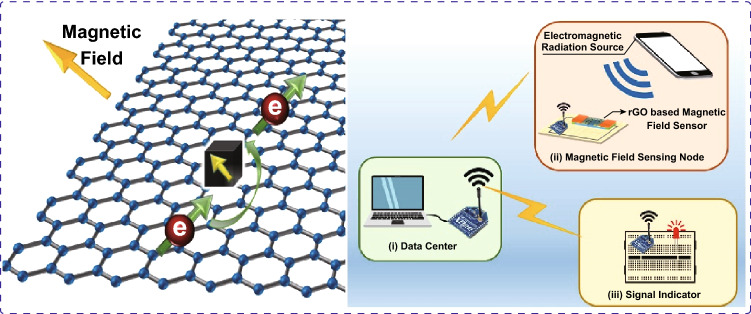

## Introduction

Magnetic field sensor is extremely useful for today’s industry for measurement and control purpose. The detectable targets for a magnetic sensor can be magnetized objects, electrical currents, or electromagnetic fields. Recent studies show that exposure to electromagnetic radiation (EMR) could be related to unfavorable health/biological effects (including carcinogenicity) on children, adults, and animals [1–4]. Concerns on low-level electromagnetic radiation exposure have been rising due to the widespread dissemination of mobile phones and other digital personal communications systems with the fast-growing wireless communications industry [5–7], whereas the lack of a sensitive sensing device for quickly detecting low-level electromagnetic fields (EMF) leads the difficulties to have information collection and decision making to the effects of EMF generated by personal electronic devices on environment and health.

Magnetoresistance (MR) refers to the change in electrical resistance of the materials under an external magnetic field. Magnetic field sensor by applying MR nanostructured materials shows significant advantages over traditional sensors. Most commercialized magnetic field sensors utilize Hall effect technique in which the output voltage on a semiconductor thin film is proportional to the strength of applied magnetic field when the current is consistent [8]. Recently, the superconducting quantum interference detectors (SQUID) and spin resonance magnetometers have been developed for detecting low magnetic field causing by artificial sources, for instance, electrical motors, high-voltage ac/dc wires [9]. Compared to these magnetic field sensors, the MR-based magnetic field sensor has the advantageous in terms of low cost, less energy consumption, physical size, and high capability in detecting low magnetic field [9, 10]. However, very few studies have reported on using a magnetic field sensor to detect or measure low-level EMR because of the lack of materials with high MR properties at the low magnetic field at room temperature. Current multilayers with giant magnetoresistance made of ferromagnetic layers separating by nonmagnetic layers require expensive manufacturing processes [11]. In addition, most developed granular nanostructured materials have lower magnetoresistance at low magnetic fields (< 10 kOe) at room temperature [12]. Recently, unusual phenomena of transport in graphene have been observed as the consequence of unusual energy dispersion relation at the Fermi energy [13, 14]. Studies have been reported that graphene with extraordinary electronic, optical and outstanding mechanical properties has been proved to lead a completely different kind of two-dimensional electronics as compared to other semiconductors [15]. Graphene-based materials have been supplied in flexible electronics for various applications including next-generation devices for diagnosis and health care [16]. MR is one of the most interesting transport phenomena in graphene, which could allow graphene-based materials to significantly contribute to the miniaturized spintronic devices [13, 14]. Many attempts have been made in developing graphene or graphene-based MR materials [17–20]. Unfortunately, all these materials show the linear MR properties, that is, their MR value at a low magnetic field (10 kOe) is quite small < 8.0% [17–20], which are not suitable for quickly sensing low-level EMR. Consequently, new manufacturing processes and suitable theory of the magnetotransport in graphene-based materials are in high demand to overcome the barriers for the development of graphene-based materials with large MR at low magnetic fields at room temperature.

The unique transport properties of two-dimensional materials are highly dependent on their defect structures [15]. Studies indicate that a reduced graphene oxide (rGO) sheet could show abnormal electrical and magnetic properties because of the altering of conducting π–π* states in a weakly disordered graphene sheet [21, 22]. In addition, incorporating inorganic elements or nanocrystals with graphene nanosheets can alter the charge carriers and scatters in graphene, which can lead to the change of magnetotransport properties [13, 20, 23, 24]. Abrikosov pointed out that large quantum MR could occur in inhomogeneous materials consisting of gapless semimetal layers with relatively small electron concentration and clusters of metallic atoms embedded in it [25, 26].

In this paper, we demonstrate that hybrid rGO nanosheets with quantum MR properties produced by a facile process have very large MR values up to 21.02 ± 5.74% at 10 kOe at room temperature. In this process, FeCo nanocrystals can in situ deposit onto the surface of rGO (FeCo/rGO hybrid nanosheets) because defects and residual functional groups on the rGO surface provide the nucleation sites for the formation of nanoparticles [27, 28]. The MR value can be tunable by adjusting the particle density of FeCo NPs on rGO nanosheets. In addition, the rGO-based MR sensor is seamlessly integrated with a wireless system to real-time detect and indicate the EMR generated by a mobile phone in a timely manner.

## Experimental

### Materials

Ferrous chloride tetrahydrate (FeCl_2_·4H_2_O, puriss. p.a., ≥ 99.0%), cobaltous acetate tetrahydrate [Co(OAc)_2_·4H_2_O, ACS reagent, ≥ 98.0%], sodium hydroxide (ACS reagent, ≥ 97.0%, pellets), and hydrazine hydrate (reagent grade, N_2_H_4_ 50–60%) were purchased from Sigma-Aldrich. Graphite flake, natural, -325 mesh, 99.8% (metals basis) was purchased from Alfa Aesar. Potassium permanganate (≥ 99.0%, ACS) was purchased from Millipore Sigma. Ethylene glycol (EG) was purchased from Fisher Chemical. Sulfuric acid (98%) and hydrochloric acid (37%) were purchased from Caledon Laboratory Chemicals.

### Preparation of Reduced Graphene Oxide

rGO was obtained by reduction of graphene oxide with hydrazine which can introduce defects and amine groups on rGO surface [29]. Graphite flake (1 g) was added to 50 mL concentrated sulfuric acid (98%). Potassium permanganate (3 g) was gradually added while maintaining the temperature of the solution below 10 °C. After proper stirring for 20 min, the suspension was kept stirring for 22 min, followed by 8 min of sonication in an ultrasonic bath (Branson 2510). The stirring–sonication process was repeated 12 times (6 h in total) before the reaction was quenched by adding 200 mL of milli-Q water into the mixture. The mixture was further sonicated for 2 h before adjusting the pH to 7 by sodium hydroxide solution (1 M). Before the reduction process, the suspension was sonicated for another hour. The reduction process was carried out by adding 50 mL of hydrazine hydrate (50–60%) solution into the mixture at ambient temperature before the temperature of the mixture increased to 90 °C for 3 h. After the mixture cooled to room temperature, the black rGO precipitates were simply collected by high-speed centrifugation and washed by 1 M hydrochloric acid. The black precipitate was in by milli-Q water and freeze-dried.

### Preparation of FeCo/rGO Hybrids

FeCo/rGO hybrid nanosheets were prepared by a modified polyol process [30]. Briefly, 2.5 mmol of ferrous chloride tetrahydrate (FeCl_2_·4H_2_O), 2.5 mmol of cobaltous acetate tetrahydrate [Co(OAc)_2_ 4H_2_O], 200 mmol of sodium hydroxide, and varied mass of rGO were added into 100 mL of ethylene glycol (EG), respectively. Five samples with different mass ratios of rGO (*M*_rGO_) added in the reaction. The samples (*M*_rGO_ increasing from 10 to 50 wt% with an interval of 10%) are denoted to Samples 1–5. After proper mixing for 20 min, the suspension was sonicated for 10–20 min to allow the rGO to disperse uniformly in ethylene glycol. The mixture was further heated up to 130 °C for 1 h under nitrogen gas protection. The black precipitate was washed by ethanol, centrifuged, and freeze-dried.

### Magnetoresistance Measurement

Each sample with 10.0 mg was pressed with a dimension of 1 × 1 cm^2^, and the thickness is kept with 150 µm. The magnetoresistance measurement of the samples was taken by Model 74046 magnetoresistance (MR) probe attached to the vibrating sample magnetometer (VSM, LakeShore 7407). In the MR measurement and mathematical calculation part, the MR (%) is defined by Eq. 1:

1 $${\text{MR}}\left( \% \right) = \frac{{R - R_{0} }}{{R_{0} }} \times 100\% .$$

The resistance change (Δ*R*, Ω) is defined by Eq. 2:

2 $$\Delta R = R - R_{0}$$

where *R* is the total resistance of the sample under magnetic field (Ω) and *R*_0_ the sample resistance at zero magnetic field (Ω).

### Material Characterization

Transmission electron microscope (TEM) micrographs and electron diffraction patterns were taken by Philips CM-10 (TEM) operating at 80 kV. A Hitachi S-3400N scanning electron microscope (SEM) attached with INCA PentaFET-x3 system (Oxford Instruments) was used to obtain SEM micrographs and energy-dispersive X-ray spectra (SEM–EDX) of the FeCo/rGO hybrids. The magnetic property and magnetoresistance measurements were performed by vibrating sample magnetometer (VSM, LakeShore 7407, moment measure range 10^−7^ to 10^3^ emu; field accuracy ‘± 0.05%’ full scale). The PHI Quantera scanning X-ray microprobe (X-ray source: Al Kα, sputter rate 2.7 nm min^−1^, takeoff angle 45°) was utilized to measure the X-ray photoelectron spectra (XPS) and depth profile of FeCo/rGO nanocomposite chips (sputter depth ≤ 81 nm). X-ray diffraction spectra (XRD) of the samples were measured by Rigaku rotating-anode X-ray diffractometer (XRD, Co-Kα radiation). The MR value as a function of magnetic field is measured at least three times for each sample.

### Software for Data Simulation

The mathematical calculation was performed by MATLAB (The MathWorks), Python(x,y) (https://python-xy.github.io/), and 1stOpt (7D-Soft High Technology Inc.), which gave us similar results.

### Wireless Magnetic Field Sensing System by Embedding the Hybrid rGO Nanosheets in the Sensor Node

The ZigBee radio module unit (XBee^®^/XBee-PRO^®^ ZigBee RF Modules) was produced by Digi International (Digi International, Inc.). All electronic devices involved were purchased from Digi-Key Electronics (https://www.digikey.com/). The magnetic field sensor made of Sample 5 with large MR value was connected with a resistor with appropriate resistance in order to perform in the physical pins’ function range of ZigBee module.

## Results and Discussion

### Development of FeCo/rGO Hybrid Nanosheets Used in the Sensor Node

rGO was obtained by reduction of graphene oxide with hydrazine which can introduce defects and amine groups on rGO surface [29]. In a modified one-pot process [30], the mixture of iron (Fe^2+^) salt and cobalt (Co^2+^) salt can be reduced to Fe^0^ and Co^0^, and FeCo nanostructures deposited on the surface of rGO nanosheets through reacting with sodium hydroxide in ethylene glycol (EG) at 130 °C in an inert (N_2_) atmosphere. Different amounts of rGO were introduced in the one-pot reaction, respectively, which were denoted as Sample 1 to Sample 5 with increasing the mass ratio of rGO (*M*_rGO_) from 10 to 50 wt% with an interval of 10%.

**Fig. 1:**
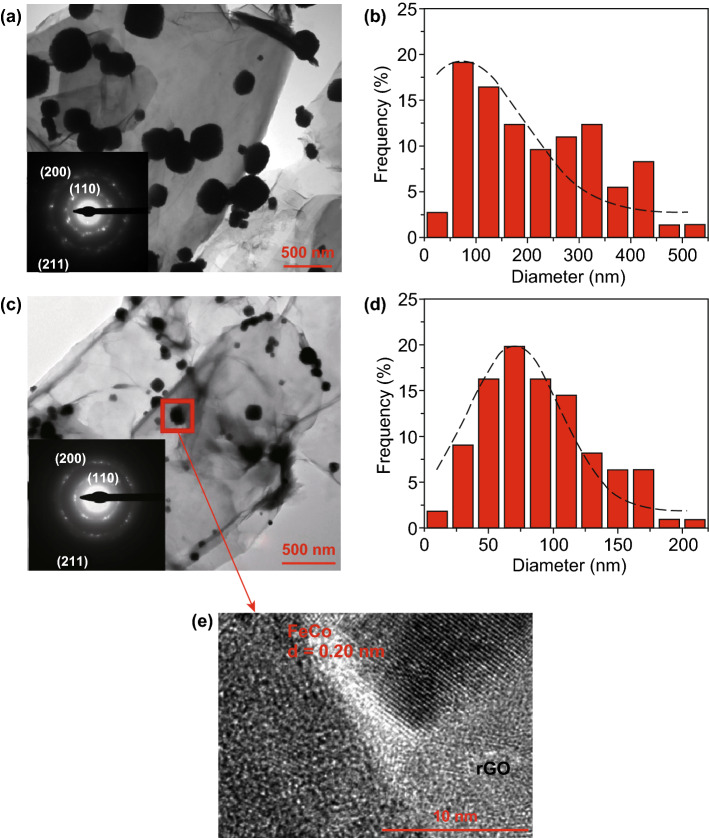
TEM micrographs of FeCo/rGO samples. **a** TEM micrographs of Sample 1 (*M*_rGO_ = 10 wt%); the small inset is the SAED pattern of Sample 1. **b** Size distribution of Sample 1. **c** TEM micrographs of Sample 5 (*M*_rGO_ = 50 wt%); the small inset is the SAED pattern of Sample 5. **d** Size distribution of Sample 5. **e** HRTEM micrograph of Sample 5

FeCo/rGO hybrid nanosheets were measured by transmission electron microscope (TEM). When *M*_rGO_ increases from 10 to 50 wt%, the average particle size of the FeCo nanoparticles (NPs) growing on the rGO is decreasing from 217 ± 50 to 89 ± 20 nm correspondingly as shown in Fig. 1. The cubic structure of nanoparticles is observed in all samples (Fig. 1a, c). The size distribution of nanoparticles is narrower when *M*_rGO_ increases to 50% (Fig. 1b, d). The decreased particle size of FeCo NPs with increasing *M*_rGO_ could be related to the increased nucleation sites on rGO as the in situ deposition occurs on the defects and residual functional groups of rGO. The selected area electron diffraction (SAED) patterns of Sample 1 and Sample 5 are shown in the inset images of Fig. 1a, c. The diffraction ring patterns indicate the body-centered cubic (BCC) structure of the FeCo bimetallic NPs with (110), (200), and (211) crystal planes. The result is similar to the reported FeCo nanostructured alloy [31, 32]. Meanwhile, all samples were measured by HRTEM. Figure 1e shows the HRTEM micrograph of Samples 5. It clearly shows the BCC FeCo nanoparticles with interplanar distance (*d*) of 0.20 nm in situ deposited on rGO on which the defects caused by the oxidization of graphene can be observed.

**Fig. 2:**
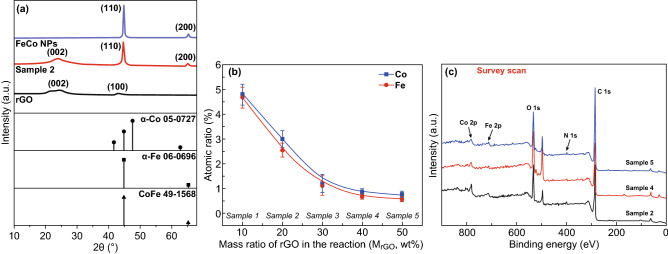
Crystal structure and chemical compositions of FeCo/rGO hybrid nanosheets. **a** XRD profile of FeCo NPs, FeCo/rGO hybrid (i.e., Sample 2, *M*_rGO_ = 20 wt%), and rGO. **b** The atomic ratio of Fe and Co with different *M*_rGO_ measured by EDX. **c** XPS survey spectra of Samples 2, 4, and 5 (i.e., *M*_rGO_ = 20 wt%, 40 wt%, and 50 wt%, respectively)

To further investigate the crystal structure of the FeCo bimetallic NPs deposited on rGO sheets, hybrid rGO samples were measured by X-ray powder diffraction (XRD) as compared to the rGO nanosheets and FeCo NPs made by the polyol process. In Fig. 2a, the XRD profile of rGO shows two broad diffraction peaks around 23.5° and 43.3° representing (002) and (100) planes of rGO, respectively. The typical peak of graphene oxide at 2*θ* = 11° in XRD profile is disappeared after the hydrazine reduction [33]. The XRD profile of FeCo NPs without rGO has two peaks centered at 44.8° and 65.3° referring to (110) and (200) crystal planes of the BCC FeCo alloy (CoFe, JCPDS No. 49-1568) [34]. Furthermore, no single Co phase is observed in the pattern. The result confirms that BCC phase of FeCo alloy is successfully produced by polyol process [34, 35]. Since all the products are stored in the ambient environment, the possibility of pure Fe metal can be eliminated [34]. Figure 2a also shows the XRD profile of the hybrid rGO, i.e., Sample 2 (*M*_rGO_ = 20%). The two diffraction peaks of (110) and (200) crystal planes of the BCC FeCo alloy are observed. The presence of the diffraction peak at 23.5° indicates that (002) plane of rGO remains in the hybrid materials; while the peak at 43.3° referring (100) plane of rGO disappears in the XRD profile of hybrid samples (see the XRD profile of Sample 2) due to the in situ growth of FeCo NPs on rGO surface which reduces the graphitization degree [36]. Thus, the in situ deposition of FeCo NPs on rGO sheets by the modified polyol process maintains the alloy structure. It is also noted that the crystal structure of the FeCo NPs deposited on rGO may change when *M*_rGO_ increased beyond 50% due to the interference of imported ions/impurities in a large amount of rGO which we will discuss in other paper.

Four major elements, i.e., C, O, Fe, and Co, are detected in all samples by the energy-dispersive X-ray spectra (EDX). The atomic ratio of bimetallic FeCo in samples decreases from 9.6 ± 0.4 to 1.3 ± 0.1 at.% when *M*_rGO_ increases from 10 to 50%. The weight percentage (wt%) of FeCo NPs in samples is estimated as shown in Fig. S1. Sample 1 (*M*_rGO_ = 10%) has 39.3 ± 2.2 wt% of FeCo NPs, and Sample 5 has 8.7 ± 1.4 wt% of FeCo NPs, though *M*_rGO_ is 50%. The exponential fitting curve (*y* = 7.9 + 31.9e^((*x*−32.1)/67.1)^, *R*^2^ = 0.965) depicts the relationship between the weight percentage of FeCo NPs deposited on rGO and *M*_rGO_ (Fig. S1). The atomic ratio of Fe:Co is approximately 1:1 to all samples as shown in Fig. 2b.

XPS survey and in-depth XPS were performed to analyze the chemical status of the major elements in FeCo/rGO hybrid nanosheets. The peaks of O 1s, C 1s, N 1s, Co 2p, and Fe 2p are observed in the XPS survey scan spectra (Fig. 2c) of Samples 2, 4, and 5 (*M*_rGO_ = 20, 40, 50 wt%). The O 1s peak indicates the presence of the remaining oxygen function groups which work as ‘anchor spot’ of FeCo NPs together with the nitrogen residues [34, 35]. The N 1s peak is caused by the hydrazine reduction process of the rGO [36], while the content (at.%) of N in samples in the samples is low, which is increased from 0.85 to 1.46% when *M*_rGO_ increased from 20 to 50%.

**Fig. 3:**
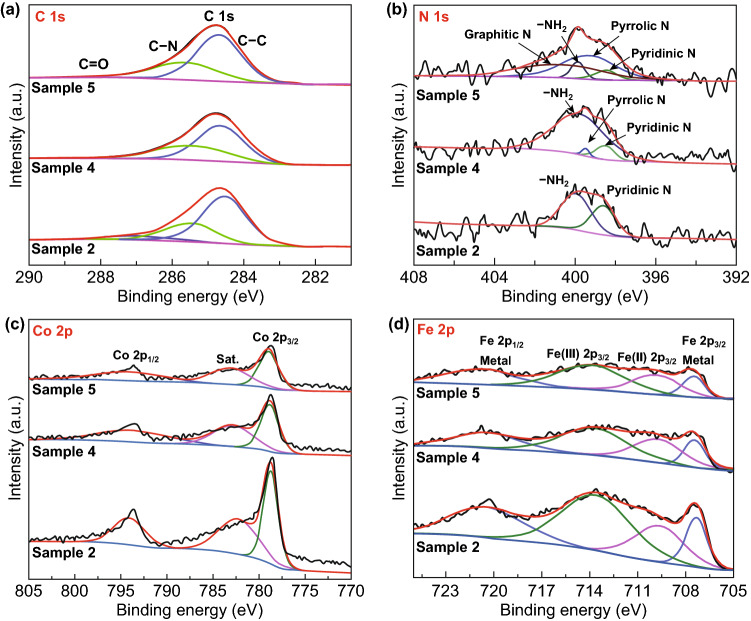
High-resolution XPS spectra of Samples 2, 4, and 5: **a** Spectra of C 1s, **b** spectra of N 1s, **c** spectra of Co 2p, and **d** spectra of Fe 2p

High-resolution XPS spectra of C 1s and N 1s are shown in Fig. 3a, b. The deconvoluted peaks of C 1s are quite similar to all samples. Three major characteristic peaks of rGO are obtained at 284.6, 285.6, and 287.2 eV which stand for C–C, C–N, and C=O bonds, respectively [37–40]. The epoxy function group is not observed in the samples due to the reduction of graphene oxide. The high-resolution N 1s XPS spectra (Fig. 3b) of Samples 2, 4, and 5 indicate the existence of pyridinic N (Sample 2: 398.6 eV, Sample 4: 398.5 eV, Sample 5: 398.4 eV) and amine groups (Sample 2: 400.1 eV, Sample 4: 400.0 eV, Sample 5: 399.9 eV), while pyrrolic N peaks are observed in the spectra of Sample 4 (399.5 eV) and Sample 5 (399.4 eV) [41, 42]. In Fig. 3b, the proportion of pyrrolic N increases, while *M*_rGO_ increases to 50 wt%. Pyrrolic N is not observed in Sample 2 which may attribute to the formation of FeCo NPs since it has a strong chelation effect with ions of Fe and Co [38]. Less *M*_rGO_ provides limited pyrrolic N spots which are almost taken by FeCo NPs during the in situ deposition, whereas the increase in rGO in the reaction introduces a large quantity of pyrrolic N acting as the nucleation site for in situ growth of FeCo NPs, leading to diameter reduction and increase in particle density of FeCo NPs on rGO. High-resolution Co 2p XPS spectra of samples are shown in Fig. 3c; peaks of Co 2p_3/2_, Co 2p_3/2_ (satellite), and Co 2p_1/2_ are found at 778.8, 782.8, and 793.5 eV for Samples 2, 4, and 5, respectively. These spin–orbit splitting peaks of Co indicate the presence of the zerovalent metallic states Co element [43]. Similarly, peaks at 707.4, 713.4, and 721.4 eV in the high-resolution Fe 2p spectra (Fig. 3d) of Samples 2, 4, and 5 are associated with Fe 2p_3/2_, Fe(III) 2p_3/2_, and Fe 2p_1/2_ [37, 44].

To further study the oxidation of Fe in samples, XPS depth profiling characterization was performed on FeCo/rGO pressed samples (Tables S1–S4). The atomic ratio of the Fe element increases with increasing sputter time, that is, the characterization depth increasing from 0 to 81 nm, while the content of the oxygen element decreases simultaneously. The atomic ratio of Fe (0) to oxidized Fe (II/III) is also increasing with increasing sputtering time (Fig. S2). Consequently, the oxidized iron may just stay on the surface because all samples are stored in the ambient environment. It is also noted that XRD provides the measure with a larger penetration depth (micrometer level), while XPS is a surface characterization. Thus, the result of XPS does not conflict with the result of XRD, indicating the formation of BCC phase of FeCo alloy on rGO. Furthermore, another interesting result is that the intensity ratio of Fe(III) 2p_3/2_ peak to Fe 2p_3/2_ peak increases with increasing *M*_rGO_. It is noted that the decrease in particle size and the increase in particle density of FeCo NPs with increasing *M*_rGO_ can enlarge the surface area of NPs; therefore, more oxidation on the surface of NPs can be detected by XPS.

### Special Magnetoresistance of FeCo/rGO Hybrid Nanosheets

**Fig. 4:**
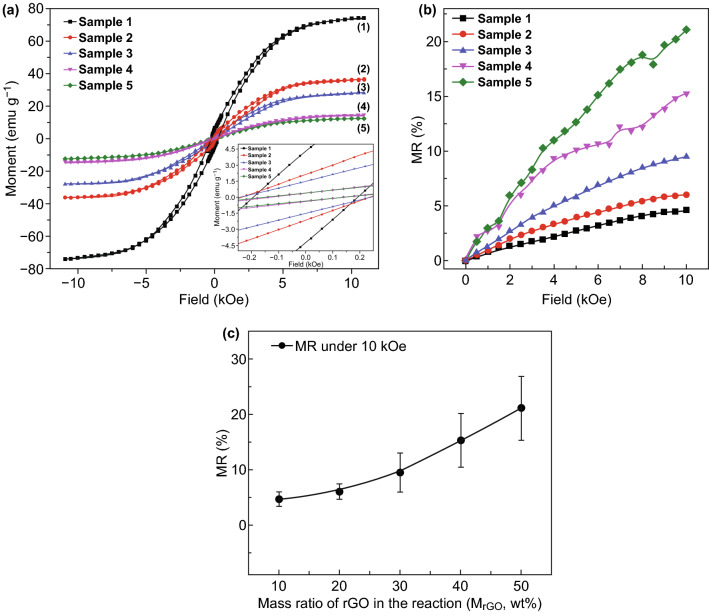
Magnetic properties of FeCo/rGO hybrid nanosheets. **a** Magnetic hysteresis loops of FeCo/rGO hybrid with different *M*_rGO_: (1) Sample 1 (*M*_rGO_ = 10 wt%), (2) Sample 2 (*M*_rGO_ = 20 wt%), (3) Sample 3 (*M*_rGO_ = 30 wt%), (4) Sample 4 (*M*_rGO_ = 40 wt%), and (5) Sample 5 (*M*_rGO_ = 50 wt%), inset is the enlarged area near field = 0. **b** MR of all 5 samples under the magnetic field of 10 kOe at room temperature. **c** MR with increasing *M*_rGO_ at 10 kOe at room temperature

The magnetic hysteresis loops of all samples were measured by vibrating sample magnetometer (VSM) at ambient temperature. The FeCo/rGO hybrids show both superparamagnetic and ferromagnetic behaviors due to the small particle size as shown in Fig. 4a. The coercivities of all samples are less than 250 Oe. The saturation magnetizations (*M*_S_) at 10 kOe for samples (*M*_rGO_ = 10–50 wt%) are 74, 36, 28, 14, and 12 emu g^−1^, respectively (Table S5). The *M*_S_ (emu g^−1^) of the FeCo/rGO hybrid decreases with increasing *M*_rGO_. All samples have a very small *M*_r_/*M*_S_ ratio which stems from the thin thickness. Meanwhile, the magnetic hysteresis loops of FeCo NPs and rGO were measured, respectively. The *M*_S_ of FeCo NPs is 181 emu g^−1^ at 10 kOe (Fig. S3a), while FeCo NPs have very small coercivity, *H*_c_ = 212 Oe, because of the small particle size. No magnetic properties can be detected to rGO at 10 kOe (Fig. S3b). To investigate the MR properties (MR (%), Δ*R/R*_0_) and further understand the relationship between MR and *M*_rGO_, the MR of Samples 1–5 was measured when the magnetic field increases from 0 to 10 kOe. In Fig. 4b, the MR value increases when the magnetic field increases from 0 to 10 kOe to all samples. The average increasing rate of MR (%) per kOe as a functional *M*_rGO_ is increasing from 0.45 ± 0.10% when *M*_rGO_ = 10 wt% to 2.50 ± 0.07% when *M*_rGO_ = 50 wt%. The *MR* value at 10 kOe increases when *M*_rGO_ increases from 10 to 50 wt% as shown in Fig. 4c. The average MR at 10 kOe for Samples 1 to 5 (*M*_rGO_ = 10–50 wt%) is 4.60 ± 1.27%, 6.03 ± 1.37%, 9.44 ± 3.52%, 15.25 ± 4.85%, and 21.02 ± 5.74%, respectively.

The quantum magnetoresistance (QMR) model proposed by Abrikosov is applied here to understand the largely enhanced MR properties of the FeCo/rGO hybrids when the magnetic field increases from 0 to 10 kOe. The QMR phenomenon in semiconductors requires that are the Landau band should be larger than the temperature and the Fermi energy in the lowest band [45]. However, Abrikosov later pointed out that in complex materials/compounds, QMR can happen when there are ‘charge reservoirs’ (atom/layer with higher electron density) embedding into the gapless layered semimetals with relatively lower electron density [25]. The electron density of FeCo is 1.1 × 10^25^ cm^−3^, and the electron density of graphene is 2.5 × 10^16^ cm^−3^ (300 K) which meets the condition calculated by Abrikosov (*n* < 10^18^ cm^−3^) [25, 46, 47]. In our assumption, FeCo NPs play the role of charge reservoirs, whereas and therefore significantly hindered the transportation of the electron carriers on rGO nanosheets [48]. As per Abrikosov’s QMR model, the total resistance of complex materials (*ρ*_*xx*_) with an excess density of carriers can be expressed by Eq. 3 [25]:

3 $$\rho_{xx} = \frac{{HN_{i} }}{{\pi ecn_{0}^{2} }} \cdot \frac{{\sinh \left( {{\raise0.7ex\hbox{$1$} \!\mathord{\left/ {\vphantom {1 \theta }}\right.\kern-0pt} \!\lower0.7ex\hbox{$\theta $}}} \right)}}{{\cosh \left( {{\raise0.7ex\hbox{$m$} \!\mathord{\left/ {\vphantom {m \theta }}\right.\kern-0pt} \!\lower0.7ex\hbox{$\theta $}}} \right) + \cosh \left( {{\raise0.7ex\hbox{$m$} \!\mathord{\left/ {\vphantom {m \theta }}\right.\kern-0pt} \!\lower0.7ex\hbox{$\theta $}}} \right)}}$$

where *n*_0_ is the excess electron density, *H* the magnetic field intensity, *N*_*i*_ the concentration of scattering centers, *e* the elementary electron charge, *c* the invariant speed, meanwhile, *θ* = *T*/*t* (*2t* is the bandwidth), *m* = *μ*/*t*, *h* = *H*/*H*_0_, and *H*_0_ = (π·*n*_0_·*c*·*d*)/*e* (*d* is the interlayer distance). The Abrikosov’s QMR model indicates that *m* = sin(π/*h*) when *T* = 0; the theoretical factor *ρ*_*xx*_ is more close to resistance change (Δ*R*) instead of actual total resistance since it equals to zero when *H* = *0* [22]. Consequently, the relationship between magnetic field intensity (*x* = *H*) and resistance change (*y* = Δ*R*) in our case can be expressed by Eq. 4:

4 $$y = b \cdot a \cdot x \cdot \left\{ {\frac{{\sinh \left( {\frac{1}{c}} \right)}}{{\cosh \left[ {\frac{{\sin \left( {{\raise0.7ex\hbox{$\pi $} \!\mathord{\left/ {\vphantom {\pi {ax}}}\right.\kern-0pt} \!\lower0.7ex\hbox{${ax}$}}} \right)}}{c}} \right] + \cosh \left[ {\frac{{\sin \left( {{\raise0.7ex\hbox{$\pi $} \!\mathord{\left/ {\vphantom {\pi {ax}}}\right.\kern-0pt} \!\lower0.7ex\hbox{${ax}$}}} \right)}}{c}} \right]}}} \right\}$$

Three different parameters (*a*, *b*, and *c*) are introduced based on Eq. 1, where *a* = 1/*H*_0_, *b* = (*N*_*i*_·*d*)/(*n*_0_·*e*^2^), and *c* = *θ*.

**Fig. 5:**
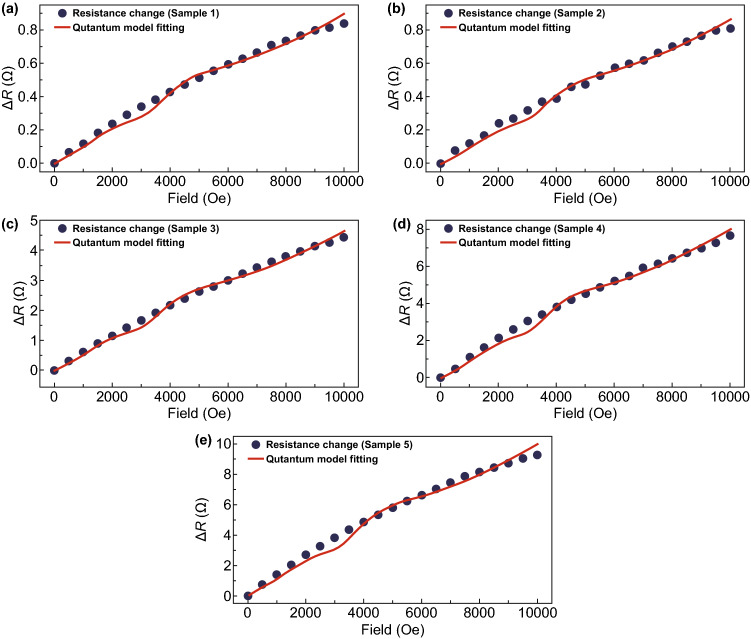
Fitting results by using Abrikosov’s quantum magnetoresistance model: **a** Sample 1 (*R*^2^ ≈ 0.996), **b** Sample 2 (*R*^2^ ≈ 0.996), **c** Sample 3 (*R*^2^ ≈ 0.997), **d** Sample 4 (*R*^2^ ≈ 0.997), and **e** Sample 5 (*R*^2^ ≈ 0.991)

**Fig. 6:**
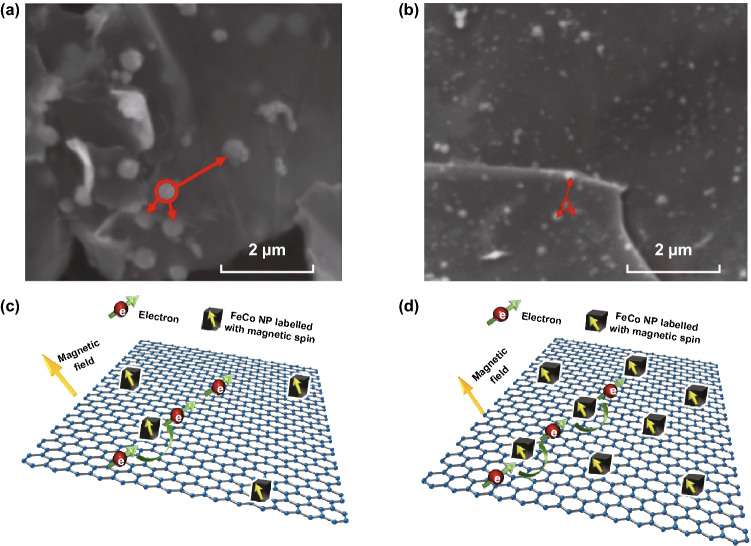
SEM micrographs of **a** Sample 1 and **b** Sample 5 (average distance for a random FeCo NPs to closest three NPs (red circle): Sample 1 ≈ 790.21 nm and Sample 5 ≈ 410.21 nm). Schematic illustration of the conduction process based on Abrikosov’s model: **c** Sample 1 with less scattering centers and **d** Sample 5 with more scattering centers. Here, FeCo NPs in situ deposited on rGO acting as the scattering centers. (Color figure online)

Abrikosov studied the different values of *θ* depending on temperature. The QMR model has been used to study different materials at different temperatures, including room temperature [17, 25, 26, 45]. Therefore, *c* is set at 1.5 for fitting optimization in our case. The experiment result shows that the resistance (Ω) of FeCo/rGO hybrid nanosheets is increasing when the magnetic field increases from 0 to 10 kOe. When *M*_rGO_ increases from 10% (Sample 1) to 50% (Sample 5), the average increasing rate in resistance per kOe is increasing from 0.0837 to 0.9282 Ω kOe^−1^ (Table S6). The fitting results are displayed in Fig. 5a–e, for Sample 1: *a* ≈ 0.0002238, *b* ≈ 1.36584 and for Sample 5: *a* ≈ 0.0002243, *b* ≈ 15.18023. Since factor *b* is relative to (*N*_*i*_·*d*)/(*n*_0_·*e*^2^), and the difference between Sample 1 and Sample 5 for factor *a* (*a* = *e*/(π·*n*_0_·*c*·*d*)) is negligible, the concentration of scattering center (*N*_*i*_) is, therefore, the dominating parameter to lead the significant increase in MR. It is noted that the particle density of in situ deposition of FeCo NPs increases as the nucleation sites of rGO increase with increasing *M*_rGO_, which also leading the reduction of particle size of FeCo NPs [48, 49]. The phenomenon is also supported by the result of TEM and scanning electron microscope (SEM). Figure 6a, b shows the SEM micrographs of Sample 1 and Sample 5, respectively. The particle density of FeCo NPs increases, while the particle size decreases when *M*_rGO_ increases.

Thus, the increase in *N*_*i*_ is directly related to the particle density of metallic nanoparticles (FeCo NPs) on rGO (Fig. 6c, d). In our study, larger particle density of FeCo NPs on rGO can have a more scattering effect. Therefore, tunable MR is easily achieved by manipulating the particle density of the scattering center, which can be controlled by varying the nucleation sites on the rGO surface.

### Wireless Magnetic Field Sensing System by Using Hybrid rGO with Special MR Properties

**Fig. 7:**
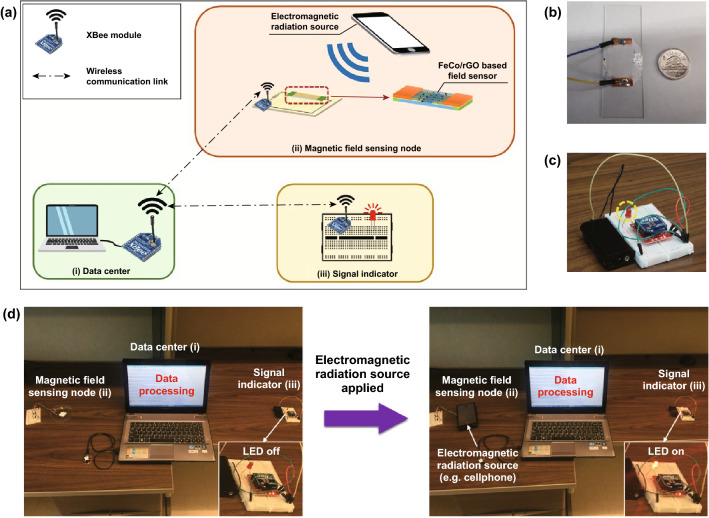
Development of the wireless magnetic field sensing and communication system with FeCo/rGO hybrid nanosheets. **a** Schematic of the wireless magnetic field sensing process. **b** Magnetic field sensor based on FeCo/rGO hybrids. **c** Signal indicator (iii) in the sensing system (LED marked in yellow circle). **d** Wireless magnetic field sensing process. While the resistance of the sensor affected by the external electromagnetic radiation source, the data center (i) receives the disturbing of voltage sent from the wireless magnetic field sensing node (ii) and sends a remote command to the wireless signal indicator (iii) to turn on the LED (small inset figure) as an alert. (Color figure online)

As shown in Fig. 5a–e, the magnetic resistance (Δ*R*, Ω) of the hybrid rGO nanosheets increases when the magnetic field increases from 0 to 10 kOe. To harness the hybrid rGO nanosheets with special MR to the real-time detection of the low electromagnetic radiation, we further integrate the magnetic field sensor made of the hybrid rGO nanosheets into a wireless communication system (Fig. 7a). Three components of the wireless magnetic field sensing system are (1) a data center, (2) a wireless rGO hybrid nanosheets-based magnetic field sensing node, and (3) a wireless signal indicator. ZigBee protocol is adopted, and three ZigBee radio modules (i.e., XBee-Pro^®^ ZigBee RF module, products of Digi International) are used for the wireless communications. More specifically, the data center (1) is formed up for data collection and visualization by linking an XBee module to the computer. The wireless magnetic field sensing node (2) is made by combining an XBee module and the FeCo/rGO hybrid nanosheets-based magnetic sensor on a motherboard. As shown in Fig. 7b, the pressed FeCo/rGO hybrid nanosheets are kept on a glass slide to easily connect with XBee module through a motherboard. The wireless signal indicator (3) is built by connecting a light-emitting diode (LED) with an XBee module (Fig. 7c). Once the resistance of the rGO-based MR sensor is affected by the external electromagnetic radiation source, the data center (1) receives the disturbing of voltage sent from the wireless magnetic field sensing node (2) and sends a remote command to the wireless signal indicator (3) to turn on the LED as an alert (Fig. 7d). The supplementary movie (Movie-S1) shows the response of the wireless magnetic field sensing system when the hybrid rGO-based magnetic sensor is approaching to an EMR generated by a working mobile phone. It is noted that the average maximum power density of a working mobile phone is in a range of 0.01–0.1 mW cm^−2^, ~ 100 nT [50]. Previous studies have shown that long-term exposure under low level (1.0–2.5 mW cm^−2^, 1 mT) EMR could lead to potential health risks [7, 51]. Our developed hybrid rGO has shown a strong change in MR when the magnetic field increases from 0 to 10 kOe (1 T); the increasing rate of resistance per kOe is up to 0.9282 Ω kOe^−1^ when *M*_rGO_ = 50%. Compared to the conventional field sensing devices, our developed wireless sensing node by using FeCo/rGO hybrid nanosheets shows the advantages of portability, real-time detection, and flexible data collection and communication.

## Conclusions

In this paper, we demonstrate a wireless magnetic field sensor made by hybrid rGO with special magnetoresistance. This wireless magnetic sensing system can quickly detect, collect data, and provide an alert signal in the presence of a low-level EMR generated by a working mobile phone. FeCo/rGO hybrid nanosheets are fabricated through a facile and cost-effective process, and large MR value is achieved, up to 21.02 ± 5.74%, at room temperature under 10 kOe, which is over two times higher than the MR values of other reported graphene-based materials at the magnetic field of 10 kOe at room temperature [17–20]. With increasing the amount of nitrogen-based functional groups on rGO, the particle size of FeCo NPs decreases, whereas the particle density of FeCo NPs in situ deposited on rGO increases due to the increase in nucleation sites. The quantum magnetoresistance of hybrid rGO nanosheets is able to be depicted by using Abrikosov’s quantum model. The large MR value is able to be controlled by the particle density of FeCo NPs acting as the electron scattering center concentration rather than their particle size. A wireless magnetic field sensing node was built by connecting FeCo/rGO hybrid nanosheets and ZigBee radio module; the increasing rate of resistance per kOe is 0.9282 Ω kOe^−1^ to the hybrid nanosheets. Our result indicates that the wireless sensor system can successfully detect the EMR generated by a working mobile phone. Consequently, the in situ deposition of FeCo NPs on rGO by the facile process can pave the path for developing large MR materials with a cost-effective process. In addition, the sensitive rGO-based magnetic sensor can be easily integrated within a wireless system for real-time detection, data collection, and alter signaling in a timely manner which will certainly benefit the future connected society.

## Electronic supplementary material

Below is the link to the electronic supplementary material.

Supplementary material 1 (MOV 6030 kb)

Supplementary material 2 (PDF 422 kb)
