# Supplementary material for: Hybrid Reduced Graphene Oxide with Special Magnetoresistance for Wireless Magnetic Field Sensor
Source: Nanomicro Lett. 2020 Mar 10;12:69. doi: 10.1007/s40820-020-0403-9 (PMC7770704; doi:10.1007/s40820-020-0403-9)
Supplement: Supplementary file 2 — Supplementary material 2 (PDF 422 kb) [file 40820_2020_403_MOESM2_ESM.pdf]

Supporting Information for

Hybrid Reduced Graphene Oxide with Special Magnetoresistance for Wireless Magnetic Field Sensor

Songlin Yang[1], Mingyan Tan[2], Tianqi Yu[3], Xu Li[2], Xianbin Wang[3], Jin Zhang[1,] *

[1]Department of Chemical and Biochemical Engineering, Western University, 1151 Richmond St., London, ON., Canada N6A 5B9

[2]Institute of Materials Research and Engineering, Agency for Science, Technology and Research, Fusionopolis Way, #08-03, Innovis, Singapore 138634, Singapore

[3]Department of Electrical and Computer Engineering, Western University, 1151 Richmond St., London, ON., Canada N6A 5B9

*Corresponding author. E-mail: jzhang@eng.uwo.ca (Jin Zhang)

Note S1 Weight Ratio of FeCo NPs in FeCo/rGO Hybrids

The weight percentage (wt%) of FeCo NPs in samples is measured by energy-dispersive X-ray spectra (EDX) as shown in Fig. S1. A fitting curve, $y = 7.9 + 31.9e^{((x-32.1)/67.1)}$ (R^2 = 0.965), is used to depict the relationship between the weight percentage of FeCo NPs deposited on rGO and M_{rGO}.

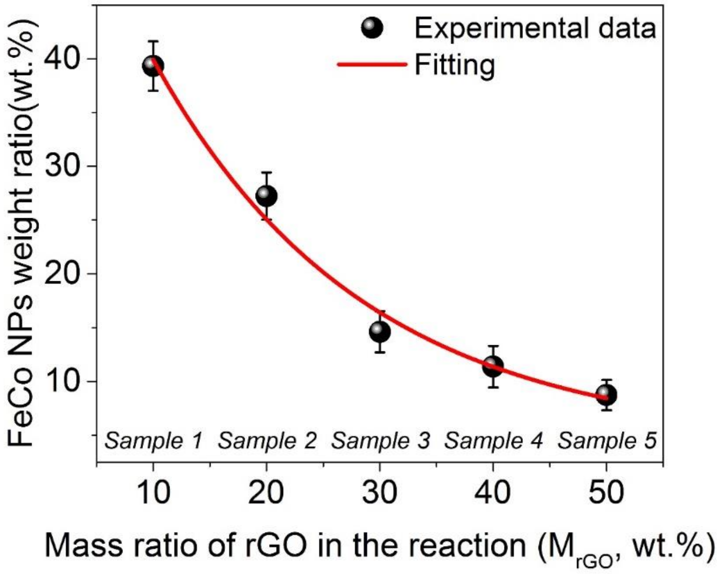

Fig. S1 Weight ratio (wt%) of FeCo NPs in FeCo/rGO hybrids (Sample 1-5 with M_{rGO} = 10-50 wt%)

Note S2 Analysis of the XPS Depth-profiling Data

X-ray photoelectron spectroscopy (XPS) depth-profiling data have been analyzed and the results are summarized in Fig. S2. The result indicates that the atomic ratio of zerovalent metallic states Fe element to oxidized Fe, i.e., Fe (II/III), is increasing with sputtering time. Table S1-S4 are the results of XPS depth profiling. The atomic ratio of Fe element in the samples increases as the characterization depth is increasing from 0 nm to 81 nm, while the content of the oxygen element decreases. Therefore, the surface oxidization under the ambient environment can be considered as the main reason based on the characterization and data analysis.

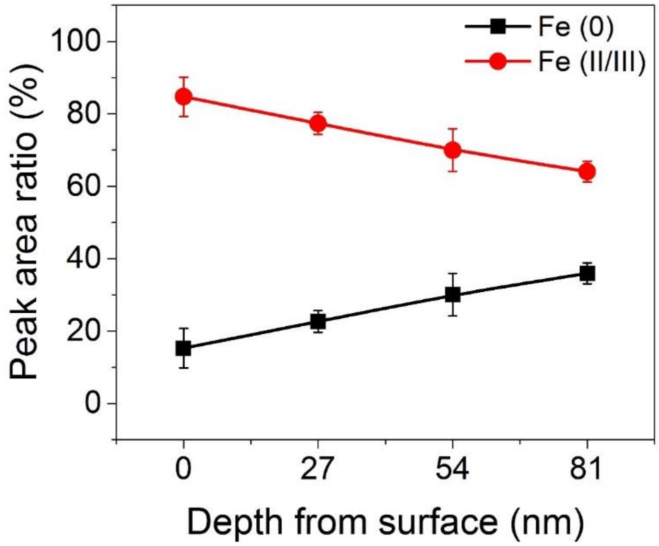

Fig. S2 XPS peak ratios of Fe and Fe (II/III) with increasing characterization depth

Table S1 XPS depth profiling results of atomic ratio of major elements of Sample 2

Sputter time (min)	Depth (nm)	C 1s	O 1s	Fe 2p	Co 2p
0	0	80.5	16.5	1.2	1.9
10	27	82.3	9.3	4.5	3.9
20	54	81.3	8.0	6.3	4.5
30	81	80.3	7.7	7.3	4.7

Table S2 XPS depth profiling results of atomic ratio of major elements of Sample 4

Sputter time (min)	Depth (nm)	C 1s	O 1s	Fe 2p	Co 2p
0	0	76.9	21.5	0.7	0.9
10	27	79.4	15.0	2.7	2.9
20	54	78.4	14.9	3.1	3.7
30	81	78.3	14.6	3.7	3.4

Table S3 XPS depth profiling results of atomic ratio of major elements of Sample 5

Sputter time (min)	Depth (nm)	C 1s	O 1s	Fe 2p	Co 2p
0	0	80.8	17.4	0.9	0.9
10	27	83.8	12.0	1.8	2.4
20	54	84.5	10.4	2.5	2.5
30	81	84.4	10.3	2.7	2.7

Table S4 Atomic ratio of iron to carbon in different samples with the increasing sputter time

Sputter time (min)	Depth (nm)	Sample 2	Sample 4	Sample 5
0	0	0.014	0.009	0.011
10	27	0.055	0.034	0.021
20	54	0.078	0.039	0.030
30	81	0.091	0.047	0.031

Note S3 Magnetic Properties of FeCo/rGO Hybrids

The magnetic properties of FeCo/rGO hybrids were measured by vibrating sample magnetometer (VSM), which shown in Table S5.

Table S5 Magnetic properties of the FeCo/rGO hybrids at room temperature

Sample No.	M_S (emu g^{-1})	M_r (emu g^{-1})	M_r/M_S	H_C (Oe)
Sample 1 (M_{rGO} 10 wt%)	74.1	4.28	0.058	192.4
Sample 2 (M_{rGO} 20 wt%)	36.4	2.15	0.059	235.2

Sample 3 (M_{rGO} 30 wt%)	27.9	1.50	0.054	233.9
Sample 4 (M_{rGO} 40 wt%)	14.2	0.38	0.027	136.6
Sample 5 (M_{rGO} 50 wt%)	12.5	0.37	0.030	138.4

In addition, the hysteresis loops of FeCo NPs and rGO were measured by VSM.

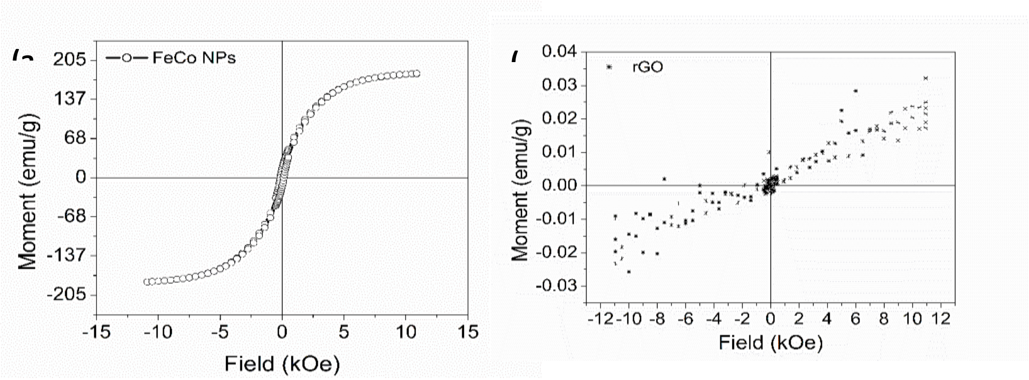

Fig S3 Magnetic hysteresis loops of (**a**) FeCo NPs and (**b**) rGO nanosheets measured by VSM

Note S4 The Increasing Rate of the Resistance of Hybrid rGO with Increasing Magnetic Field

The magnetic resistance of FeCo/rGO hybrid nanosheets increases when the magnetic field increases from 0 to 10 kOe. Table S6 lists the average increasing rate of the resistance of samples with the increasing magnetic field from 0 to 1 kOe. Please note Sample 1 refers M_{rGO} = 10 wt%, Sample 2 (M_{rGO} = 20 wt%), Sample 3 (M_{rGO} = 30 wt%), Sample 4 (M_{rGO} = 40 wt%), and Sample 5 (M_{rGO} = 50 wt%).

Table S6 The sensitivity of the magnetic field sensors made of hybrid rGO nanosheets to the low magnetic field (< 10 kOe) at room temperature

Sample No.	Sample 1	Sample 2	Sample 3	Sample 4	Sample 5
Ω kOe^{-1}	0.0837	0.0857	0.4431	0.7660	0.9282

Supplementary Movies

Moive-S1 Demonstration of the wireless magnetic field sensing process by integration of hybrid rGO-based MR sensor and XBee module
